# Advance care planning in German nursing homes from the perspective of the facilitators: A focus group study

**DOI:** 10.1186/s12904-025-01914-z

**Published:** 2025-10-15

**Authors:** Anna Völkel, Nadja Reeck, Tanja Schleef, Hannes Jacobs, Stephanie Stiel, Falk Hoffmann, Anna Levke Brütt

**Affiliations:** 1 Junior Research Group for Rehabilitation Sciences, Department of Health Services Research, Carl von Ossietzky Universität Oldenburg, Oldenburg, Germany; 2 University of Applied Sciences and Arts Hildesheim, Faculty of Social Work and Health, Hildesheim, Germany; 3 Institute for General Practice and Palliative Care, Hannover Medical School, Hannover, Germany; 4 Division of Outpatient Care and Pharmacoepidemiology, Department of Health Services Research, Carl von Ossietzky Universität Oldenburg, Oldenburg, Germany; 5 University Medical Center Hamburg-Eppendorf, Department of Medical Psychology, Hamburg, Germany

**Keywords:** Advance care planning, Nursing home, Focus group, Advance care planning facilitators, End-of-life care

## Abstract

**Background:**

Since 2018, German nursing homes have been able to reimburse advance care planning (ACP) at the expense of statutory health insurance. ACP is a consultation for end-of-life care in which care preferences can be documented. The consultation is conducted by facilitators, who have completed the required training. However, limited research exists on how the ACP consultation processes are realized. Hence, this focus group study, as a part of the “Gut-Leben” research project, investigated its implementation.

**Methods:**

Twenty-four ACP facilitators participated in four semi-structured focus groups conducted between July and September 2023. The first three focus groups were held in person with participants from Lower Saxony and Bremen, and the fourth was conducted digitally to include facilitators from other federal states in Germany. The interview guide was developed with the project’s practice advisory board in advance. The analysis was performed using deductive-inductive content analysis based on Kuckartz and Rädiker.

**Results:**

The facilitators’ average age was 51.7 years (range 30–70), with 75.0% being female (*n* = 18). Facilitators typically reached out to residents proactively with the support of the nursing staff, who acted as intermediaries and helped to establish contact between the facilitators and the residents and/or relatives. Residents and relatives rarely approached the facilitators. The ACP consultation process varied in length and frequency, beginning with an initial information meeting and followed by further meetings if needed. Update meetings could be scheduled at any time, particularly in response to changes in residents’ preferences or health status, but were implemented frequently. The consultation process and the documents created during the consultation, above all the living wills, were described as very complex, especially for cognitively impaired people, which is why standardized and simplified documents are desirable.

**Conclusions:**

The results indicate that ACP is highly individualized, varying by resident. However, there is a rough standardized procedure for the process, which, like the documents, could be very complex. ACP must be more widely promoted to raise awareness, reduce inhibitions, and simplify the initiation of consultation processes. Besides, ACP must also be integrated into the structure of nursing homes as a fixed procedure.

**Supplementary Information:**

The online version contains supplementary material available at 10.1186/s12904-025-01914-z.

## Background

Nursing home (NH) residents are often in the last phase of their lives when they enter the NH, which are nowadays considered to be a place for dying [1–3]. Dignified dying is essential for proper end-of-life (EOL) care, primarily defined by the resident’s desire for autonomy [4, 5]. However, cognitive impairment or dementia can limit the ability to make decisions and express oneself, so that individual wishes and preferences for medical and nursing care in the last phase of life can no longer be communicated [6, 7]. Advance care documents, such as a living will, can record these preferences at an early stage in case they can no longer be communicated [8].

Anglo-American countries consequently introduced the concept of advance care planning (ACP) in the 1990 s to document EOL wishes, including preferences for medical and nursing measures or for refusing them, in case the individual is unable to provide consent [9–11]. Based on the ACP concept, § 132 g Social Code Book Five (SGB V: Health Care Planning for the Last Phase of Life) was introduced in Germany in 2018 allowing NHs to provide consulting services based on ACP and to be reimbursed by statutory health insurances. The consultations are provided by ACP facilitators who have completed specific training in accordance with § 132 g SGB V [12]. The prerequisite for participation in the training to become an ACP facilitator is completion of vocational training, e.g. as a nurse, or a degree in the field of health and nursing sciences. Additionally, it requires three years of professional experience relevant to health care planning within the last eight years, covering at least half a position and completed in an inpatient or outpatient care facility [12]. In addition to providing a qualified facilitator, NHs must also submit a palliative care concept and a plan for integrating ACP into the facility structure in order to receive the relevant approval [12].

Through structured consultations with trained ACP facilitators, NH residents can discuss their medical and nursing preferences for EOL care [12]. These preferences can be documented in advance care documents, such as living wills [12]. ACP consultations should not be seen as a one-off service that necessarily results in the creation of care documents. Rather, such a process can be restarted at any time if, for example, the wishes or state of health of the resident changes, meaning that documents that have already been drawn up can be revised again [12].

However, although the respective § 132 g SGB V was already introduced in 2018, only around 15% of German NHs were offering the ACP service at the turn of the year 2022/2023 [13]. In addition, little research has been carried out into how ACP is implemented in Germany in accordance with § 132 g SGB V.

The following research question was thus investigated: “How is ACP implemented in NHs in Germany from the perspective of the ACP facilitators, and what barriers and support factors affect its implementation?”. This may allow us to gain an insight into practices and to identify improvement strategies.

## Methods

### Design

This focus group study is part of the Gut-Leben project: *Implementation*,* barriers*,* and recommendations for further development of advance care planning for the last phase of life in nursing homes in Germany (Gut-Leben)*. Using a mixed-method approach, the project examines the implementation of legally mandated EOL healthcare planning options established by § 132 g SGB V in 2018 [14]. Additionally, the Gut-Leben project is supported by a practice advisory board that provides guidance to the study team throughout the entire project (for example developing survey instruments or discussing results). The practice advisory board consists of ACP facilitators, who are predominantly trained nurses, and facility managers working in NHs where ACP is implemented.

The present focus group study heard from facilitators, to capture their perspectives on ACP practice and implementation. Semi-structured focus groups were thus conducted.

Reporting followed the COREQ checklist [15]. The Medical Ethics Committee of Carl von Ossietzky University of Oldenburg approved the study (number: 2023-071). All participants gave informed consent.

### Recruitment

From May to September 2023, ACP facilitators were recruited to four focus groups. Recruitment took place at an ACP networking meeting in Hannover (state capital of Lower Saxony) and through e-mail lists from hospice and palliative care associations, institutions offering further education and training in hospice and palliative care, organizations training ACP facilitators, all based in Lower Saxony, and the ACP Germany association. The practice advisory board of the Gut-Leben project also facilitated contact. Interested participants were contacted by the research team directly by telephone, email, or in person, or could initiate contact with the study team by themselves. ACP facilitators who had completed training in accordance with § 132 g SGB V and who work or have worked in NHs were included. Participants from Lower Saxony and Bremen were recruited to the first three focus groups, while the final group was open to interested ACP facilitators nationwide. Sampling aimed to include a heterogeneous group of participants (age, sex). At the same time, variation in the NHs (size, sponsorship) in which the facilitators worked was considered.

### Data collection

The focus group guide was developed for the study by the research team in collaboration with the Gut-Leben project’s practice advisory board. First, the research team considered topics for the guide and prepared questions addressing the research question. In a second step, the draft of the focus group guide was discussed with the members of the practice advisory board. On the basis of this, the guide was adapted to the language used in ACP practice, additional topics for the focus group guide were discussed, and the content sections were structured. In the end, the focus group guide covered three content sections: (1) the ACP process, (2) success factors and barriers, and (3) suggestions for improvement (see supplemental material, *guiding questions for the focus groups*).

The four focus groups were conducted from July to September 2023, with an average duration of 115 min (range: 109 to 120 min). The first three focus groups took place in person at the Carl von Ossietzky University of Oldenburg, while the fourth was held digitally using the university’s web conferencing platform. Seven facilitators took part in the first focus group, six in the second, three in the third, and eight in the fourth.

With participant consent, the focus groups were audio-recorded and transcribed verbatim by an external professional transcription service following Fuß and Karbach [16]. All transcripts were pseudonymized.

### Data analysis

The focus groups were analysed, employing a deductive-inductive content analysis approach based on Kuckartz & Rädiker [17]. MAXQDA 24 software was used by the researchers [18]. At least two researchers (AV & NR) were involved in each analysis step, with a third researcher (ALB) consulted to resolve coding discrepancies.

The work steps were as follows: The first step was based on the focus group guide, which included three subject areas (1. description of the ACP daily care routine and the ACP processes, 2. supporting factors and barriers, and 3. improvement suggestions) and the research question, preliminary main deductive categories were identified. Second, three of the four focus groups were coded jointly by the two coders (AV & NR) using the deductive categories. Further main categories were also derived inductively from the material. Parallel to the joint coding, a category system with the main categories was developed. In a third step, this preliminary main category system was then applied to the fourth focus group with both coders (AV & NR) coding this focus group separately. Divergent coding was then discussed in a consensus meeting. Fourth, subcategories were developed inductively from the data by discussing three focus groups together again (AV & NR) and developing subcategories. A preliminary category system consisting of the main categories and subcategories was then discussed. In the fifth step, the preliminary category system was applied to the last focus group. The two coders (AV & NR) again proceeded separately with the coding. In a final consensus meeting, deviating coding was discussed and the category system was finalized. Finally, the category system included definitions and descriptions of the categories and anchor examples. The categories formed explain the course of the consultation process on the one hand and also describe the participating ACP facilitators on the other (Table 1).

**Table 1:** Overview of the focus group categories

	Categories	
Main categories	ACP facilitators	Consultation process
Subcategories	Career path Job description	Getting in touch Conversation procedure Documentation Billing

## Results

### Participants

The twenty-four participating ACP facilitators had a mean age of 51.7 years (range 30–70) and 75% were female (*n* = 18). The NHs in which the ACP facilitators worked were 58.3% non-profit (*n* = 14), 16.7% private (*n* = 4), and 4.2% (*n* = 1) public (not available *n* = 5), and have a median of 188 beds, meaning the ACP facilitators work in NHs of varying sizes.

### ACP facilitators

#### Career path

The ACP facilitators completed their ACP training in the period from 2017 to 2022, so they have been working as ACP facilitators for different lengths of time.

*“I did the ACP training as an ACP facilitator in 2022.” [FG4]*.

#### Job description

In addition, the facilitators reported that most of them work part-time, with hours varying based on the facility’s size. Some facilitators hold additional roles in nursing, management (residential, nursing, or NH management), hospices, and palliative care services. Almost all of them call themselves “solitary fighters”, meaning they are the only facilitators working in the NH and have no facilitator colleagues.

*“I used to have a colleague*,* but unfortunately*,* yeah*,* she kinda disappeared. I have no idea what happened to her. So now*,* I am a solitary fighter*,* so to speak.” [FG2]*.

The majority are employed directly by the NH, while some work externally, meaning that the facilitators are not employed within the NH, but come into the facility from another provider.

*“Yeah*,* I actually work full-time as a nurse in elderly care*,* in a nursing home*,* during the day shift. And then I have a certain hourly rate per hour [proportion of working hours] for ACP.” [FG2]*.

### Characteristics of the consultation process

**Fig. 1:**
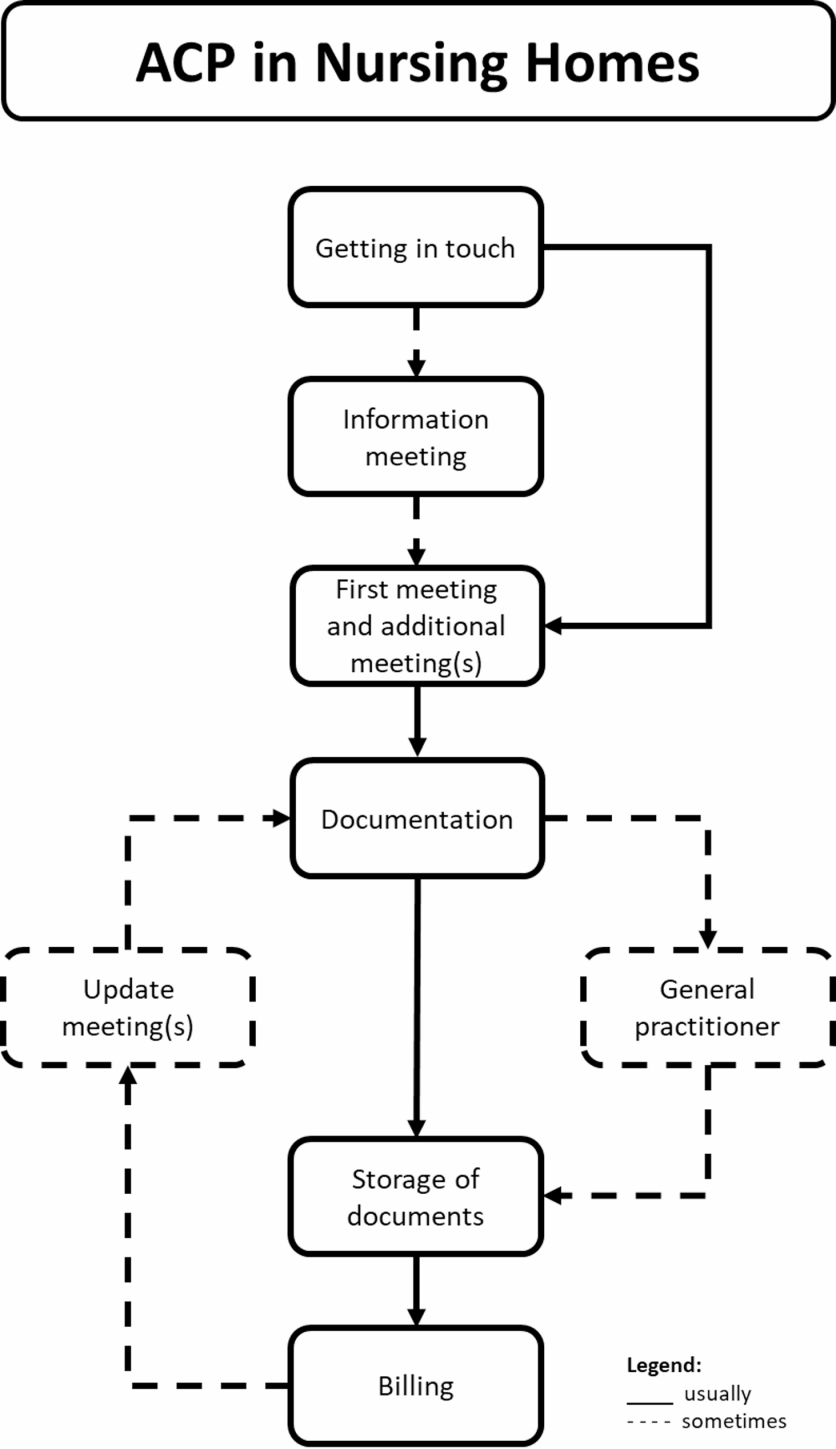
ACP consultation processes in German nursing homes

In the focus groups, the facilitators dealt with the consultation process. Despite various different experiences, the consultation process usually follows a rough pattern reported by all facilitators: it begins with the initiation, the conversation procedure with the meetings, documentation in combination with the involvement of the general practitioner (GP), the storage of the resulting documents up to billing, and possible update meetings (Fig. 1).

In addition to the described ACP process, the facilitators also discussed barriers and supporting factors for the elements of the ACP process.

#### Getting in touch

The facilitators reported that ACP the consultation process is initiated in different non-systematic ways. Various print media, including flyers, posters, brochures, letters, notices, and websites are used to promote the ACP services. Furthermore, information sessions are held at some NHs to raise awareness of the service. However, the advertising of the ACP offer is handled very differently in the NHs.

*“I also have flyers that are placed in the facilities*,* which are given to residents upon arrival.” [FG2]*.

*“Anyway*,* through an information evening.” [FG3]*.

In some cases, residents and/or relatives approach the ACP facilitators if they have previous personal experience with the dying process or if the facilitator is already known to them. Typically, ACP facilitators take an outreach, proactive approach, this being described as less systematic and more needs-oriented. They use care documentation to identify consultation needs, such as for new residents without a living will or when a resident’s condition deteriorated. The facilitators then initiate a consultation process if the residents want it, because ACP is voluntary and not a mandatory offer.

*“And then for me*,* it also means being proactive with the residents*,* the family members*,* or whoever is responsible*,* starting the conversation.” [FG3]*.

Beyond that, nursing staff, as well as administration and (palliative care) physicians, act as intermediaries by identifying potential needs and enabling contact with the facilitators.

Moreover, facilitators reported that initiation depends on how well known the consultants are. It could be challenging when residents approach facilitators only if they are already known, such as when a facilitator also works as a nurse. This makes it more difficult for new unknown facilitators to establish contact.

*“Since I’ve been working in this field for almost thirty years*,* most of the residents know me. […] They came to me because they knew me. My colleague*,* she was new*,* she just started*,* and she had a really hard time with that*,* you know?” [FG2]*.

The facilitators’ idea that the size of the NH also makes a difference accords with the above. Smaller NHs are supposedly more informal, which makes it easier to establish contact because the facilitators and the residents know each other.

Furthermore, the facilitators explained that COVID-19 has a conducive and inhibiting impact. On the one hand, it is been seen as a barrier to establishing contact, as consultations were impossible because personal contact was avoided. On the other hand, COVID-19 also acted as a catalyst for increased requests for ACP services, as the relevance of ACP was highlighted by the deaths caused by the virus.

*“That really woke a lot of people up. Because with the first wave*,* we actually didn’t have any COVID cases. We were really*,* really lucky. It was only after the fourth vaccination that we had COVID-19 patients*,* but in a nearby home*,* many residents passed away. Thirteen*,* fourteen. […] That was really bad. So*,* the family members literally stormed the place.” [FG3]*.

Another topic discussed by the facilitators was the consideration of the extent to which there is a need for such an advisory service for the residents. There were different opinions on this because the situation varies from facility to facility. On the one hand, an urgent need is seen. On the other hand, a low level of uptake is perceived in some cases. One possible reason, according to the facilitators, is that talking about the last phase of life and dying may still be a taboo, which is why the ACP consultation is probably not used. Not making use of ACP consultation causes concern among facilitators.

*“And I’m really scared that*,* since so few people take advantage of it*,* they’ll [Note: health insurance companies] say*,* ‘We don’t need this at all.” [FG 2]*.

#### Conversation procedure

The facilitators also described the conversation procedure. Once contact has been made, and if the resident wishes to receive a counselling session, mostly but not always an initial information meeting is held to explain the consulting services. This is followed by one or more additional meetings that explore how the person in question likes to live and what a good life means for the resident. Medical and nursing measures and specific treatment wishes are also clarified. Topics include the funeral as well as personal rituals and habits that are desired for the last phase of life. Overall, however, the content of the discussions varies depending on the people being advised.

*“And then*,* from the conversations*,* specific treatment wishes come out. This can*,* on the one hand*,* really focus on the medical aspects… And on the other hand*,* it can also be concrete wishes*,* like how the person wants to be cared for when they’re seriously ill or when it’s really near the end of their life. What’s important then? On the care side*,* but also things like whether they prefer the window open or closed*,* whether a lot of people should visit or not. And what’s important to the person in terms of personal rituals and habits.” [FG4]*.

The meetings typically take place in the resident’s room. If the resident is able to give consent, discussions are held solely with them, and relatives are only involved if desired. The nursing staff is rarely present. If the resident lacks the capacity to consent, discussions are held with the legal representatives, though relatives may have also been included depending on the resident’s state of health.

*”So*,* I mainly have the conversations directly with the resident. Sometimes they say*,* like*,* ‘my son’ or someone should be there. That’s fine with me. Otherwise*,* I often finish the conversations alone with the resident.” [FG2]*.

The consultations are very individual depending on the resident. It is therefore difficult for the ACP facilitators to give a general indication of the frequency and duration of the meetings and the entire consultation process. The facilitators reported that the consultation process could involve one or two meetings independent of the first short information meeting, but sometimes several meetings are needed. Individual meetings typically last between fifteen and ninety minutes, with exceptional cases lasting several hours. The consultation process could be completed within a few weeks, though it could extend for months if multiple meetings are required.

*“Sometimes there are things/residents where you have to go five or six times. With some*,* after just two times*,* it’s clear and solid on paper*,* you know?” [FG2]*.

In this respect, the type of document created could be used as a yardstick. A living will generally takes longer than an emergency form. Irrespective of the type of document to be prepared, it is more difficult to estimate the time required when legal representatives are involved.

In addition to the described process, the facilitators also reported other challenges during the actual ACP conversations. Some consultations are perceived as very complex, requiring multiple consultations to manage the flow of information and prevent the residents from becoming overwhelmed. Consultations are especially challenging for individuals with dementia and cognitive impairment, often necessitating more time.

In addition to the usual conversation process, there is also the option of an update meeting at any time, so that a new consultation process could be initiated if the circumstances, state of health, or wishes of the person concerned change. The facilitators emphasize the possibility of update meetings, which highlight the evolving nature of ACPs, allowing changes at any time.

#### Documentation

The facilitators reported that as soon as the first or subsequent meetings have taken place, they documented the results of these meetings. Living wills and emergency forms are usually mainly drawn up for the first time, or sometimes existing living wills drawn up before entering the NH are revised. Once the consultations are completed and the documents are created, they are sent to the resident’s GP.

*“And then the document goes to the general practitioner. Then I have a brief chat with her*,* so to speak*,* and then she stamps it and signs it.” [FG1]*.

The original copies of the finalized and signed documents are sent to the residents or their relatives. In every NH where the facilitators work, without exception, further copies are stored in the resident’s file and care documentation system, allowing copies to be automatically printed in times of crisis or emergencies.

*“And then I upload it into the system. Of course*,* the original goes back to the person. A copy goes to the care department*,* and a scanned file is sent to the administration*,* who then upload it into this SIS [Note: abbreviation for Structured Information Collection*,* care documentation system].” [FG1]*.

Additionally, the facilitators report on the different ways in which the emergency form is kept for emergencies. In some cases, the emergency form is also placed inside the door of the resident’s wardrobe. Additionally, some facilities use a traffic light system with dots in the residents’ rooms to indicate which measures are still desired or not desired.

*“This [Note: emergency form] is in the resident’s room*,* in the cabinet.” [FG2]*.

Similarly, the complexity of the conversations is described as challenging. There were differing opinions of the facilitators about the comprehensibility and scope of the documents produced. Some find the documents easy to understand. Others would prefer living wills in plain language. One criticism is that the ACP documents are too complex and extensive and should be simplified. Moreover, the facilitators wanted documents to be standardized.

*“Nineteen pages*,* my colleague complains every time she has to print a transfer form for the hospital. (speaks with a changed voice) ‘I don’t have that much paper in there*,* and it takes so long.’ They don’t want to wait that long. We need to find more practical solutions.” [FG1]*.

#### Billing

At the end of the consultation process, the intention for service records is to be created, collected, and sent to the health insurance providers, but this is not obligatory. The service records contain details of the insured person, the start and end of the ACP process, and the number of consultations, according to the facilitators. As the service records are not relevant for billing, not all facilitators complete and send the service certificates to the health insurance companies. Otherwise, no attention is paid to the service records issued and the associated workload because either there is no contact person at the health insurance companies, the employees of the health insurance companies do not know about ACP, or the service records are sent back.

*“For example*,* that was a problem when you don’t have a contact person. Then I once tried to call an insurance company*,* you can take any as an example*,* it doesn’t matter: 'No*,* I don’t know it. Never heard of it'.” [FG1]*.

*“I also had the experience*,* so we had agreed on the lump sum remuneration for 2020. During the first conversations*,* I filled out the service records. Sent them off. Got no feedback. I contacted them via email*,* the person had no idea*,* because it’s actually under § 132 g*,* and we don’t need it. You get a lump sum remuneration.” [FG1]*.

## Discussion

This focus group study explored the implementation of ACP consultation processes from the perspective of the facilitators. Overall, the facilitators outlined a similar flow for the ACP consultation, with three key points identified in the process:

1. the proactive outreach structure and the associated uptake of the counselling process,
2. the individuality of the consultation process and the relevance of the process character and
3. the complexity of the conversation and documents produced.

### 1. Proactive outreach structure and use of the ACP service

The promotion of the ACP offer, and the resulting contact is not very systematic. Contact is making predominantly in an outreach, proactive way on the part of the facilitators, and dependent on the structures present in the facilities, including available resources for nurses and the cooperation between facilitators and nurses, which play a key role in facilitating contact and supporting the ACP advice. That aligns with other studies highlighting the role of healthcare professionals in initiating and conducting ACP consultation [19, 20].

Reasons for the unsystematic approach and the outreach structure could be due to a lack of interest or willingness from residents or relatives to participate. However, in our study, facilitators reported that once contact is made, conversations usually occur, suggesting that lack of interest is not a fundamental issue. Rather, it could be that there is a lack of awareness about available counselling services [21]. This may be due to insufficient advertising and information campaigns or a lack of knowledge about the facilitators [20–23], and explained why residents and relatives are less likely to approach facilitators on their own. Furthermore, advertising the offer could reduce the taboo around dying and death that may contribute to the underuse of ACP services [24, 25], as the residents avoid ACP conversations about EOL issues due to discomfort [19, 26].

Furthermore, the COVID-19 pandemic, as outlined by the National Association of Statutory Health Insurance Funds in its report on palliative care (2022), impacted ACP implementation [13]. During contact restrictions and fear of infection, counselling was not often possible, reducing the use of ACP consultation [27] and making implementation more difficult, although the facilitators noted that COVID-19 acted as a magnifier, highlighting the relevance of ACP. Hartog et al. (2020) also confirmed that the pandemic increased the need for ACP [28]. However, the more difficult implementation due to COVID-19 could also mean that the ACP structures in the facilities could not be established. Furthermore, being a “solitary fighter” is a challenge to ACP implementation. As reported by the facilitators in our study, other studies found that there was predominantly only one facilitator working in the facilities [29, 30]. This means that counselling sessions have to be cancelled in the event of illness or vacation, and are not taken over by colleagues. Pool solutions could help here [31]. Being a “solitary fighter” and the challenging nature of establishing contact may indicate that ACP is not well integrated into the facility’s overall workflow. ACP and its facilitators should be part of a comprehensive palliative care concept within the NH structure.

### 2. Individuality of the discussion processes and the nature of the process

Although ACP processes and their structure are reported to be fundamentally similar, the number and duration of meetings as well as the duration of the entire counselling process vary greatly. This could be explained by the very individual nature of EOL care and the correspondingly different content of the consultations [32], also depending on the needs of residents and relatives. However, individuality also means that processes are less structured and may deviate from the intended standardized procedure for the process. This may lead to few or no update meetings, meaning that the nature of the process, which by definition is intended for ACP [12], seems to get a bit lost. Likewise, Jacobs et al. (2024) also found that few update meetings were recorded [29]. One reason might be that update meetings are voluntary. Secondly, further update meetings might not be desired because of the difficult topic [24, 25]. Another reason could be a lack of knowledge about this option or a lack of interest on the part of residents and relatives [21–24]. Furthermore, the facilitators’ lack of time for updating [33] might play a role. One reason for the lack of time could be that the facilitators have to spend a lot of time on an initial consultation process. The lack of time may also result in a selective approach, especially neglecting meetings with residents who already bring documents with them to the facility. However, in these cases update meetings could be most important, because this subgroup is and was already interested in ACP. Furthermore, a transfer to an NH is often accompanied by a deterioration where preferences can also change and, consequently, documents brought along by residents need to be updated.

### 3. Complexity of conversations and documents

The third key point is that the conversations and, in particular, the documents were predominantly assessed as too complex by the ACP facilitators, especially for cognitively impaired residents. These empirical values are followed by the consideration of whether ACP is suitable for NHs if a growing proportion of NH residents is already affected by dementia [34, 35]. The complex ACP conversations and documents require adaptation, especially for people with dementia, which could also prove helpful for the implementation of ACP conversations and the creation of documents [36, 37]. As a consequence, this group of people can also be given more self-determination [37].

The complexity of ACP processes also helps explain why they are so individualized. Healthier residents may find it easier to engage in these conversations, resulting in shorter durations and fewer meetings compared with those who may have cognitive impairment, requiring more time. Simplifying the documents could reduce the variability in conversations, making them more structured and easier to implement. Additionally, this simplification could increase service uptake by removing the complexity that puts resident off, allowing even those with cognitive impairment to access the conversations more easily.

### Strengths and limitations

This study makes a significant contribution to providing insights into the processes of ACP, which have been insufficiently investigated and reported to date, and to reviewing the relatively new, legally anchored possibility of billing ACP at the expense of health insurance companies. This can support the evidence-based nature of the legislation. Another strength is the heterogeneous group of facilitators and its facilities: they spend varying amounts of time on ACP consultations depending on the size of the facility [12]. In this way, we include facilities of different sizes and also reflect the typical picture of the German care home landscape with the sponsorship of the facilities, which are predominantly non-profit [38]. In addition, the digital focus group broadens the perspective beyond Lower Saxony and Bremen and allows the assumption that the results reflect the implementation of ACP throughout Germany, as similar discussions were held in all focus groups, the digital one and the face-to-face ones. This strengthens the relevance and generalization of the results for the whole of Germany.

Despite these strengths, there are several limitations that restrict the generalizability of the results. One key aspect is the international comparability of the results. As the implementation of ACP in care facilities is at different stages in other countries [39–47], the study cannot make any statements about the extent to which the practical examples presented can be transferred to other countries in terms of ACP implementation. However, fundamental considerations relating to the process can be derived, for example, that the documents used and to be created should be adapted to the target group.

As a further limitation, it should be noted that the study does allow us to illustrate what happens when ACP is implemented. However, we cannot make any statements about the effect and outcome of ACP. Further sub-studies in the project will evaluate this [14].

Finally, the study may contain a selection bias, as those involved already have experience with ACP implementation and the necessary structures in place. As a result, it may not fully capture the challenges faced by facilities lacking these structures. However, the facilitators still reported various challenges, highlighting the relevance of the results despite this selection bias.

## Conclusion

The ACP service and facilitators need to be better known in NHs to increase awareness and reduce hesitation in using the ACP service. Additionally, the conversations and documents, often perceived as complex, should be simplified, especially for people with cognitive impairment, in order to provide this group of people with an ACP service as well. ACP and the facilitator must also be better integrated into the structures and processes in the facilities as part of an overall palliative concept to counteract the existence of “solitary fighters”. Besides, the consultation process must be initiated directly and systematically on admission to the NH. Further, it makes sense to shed light on further perspectives on the implementation of ACP and its effects.

## Supplementary Information

Supplementary Material 1.

Supplementary Material 2.

Supplementary Material 3.

Supplementary Material 4.

## Data Availability

The datasets used and/or analysed during the current study are not publicly available to protect participants privacy. Selected datasets are available from the corresponding author upon reasonable request.

## Abbreviations

ACP: Advance care planning
EOL: End of life
GP: General practitioner
Gut-Leben: Implementation, barriers, and recommendations for further development of advance care planning for the last phase of life in nursing homes in Germany
NH(s): Nursing home(s)
SGB V: Social Code Book Five
