# Supplementary material for: Advance care planning in German nursing homes from the perspective of the facilitators: A focus group study
Source: BMC Palliat Care. 2025 Oct 15;24:258. doi: 10.1186/s12904-025-01914-z (PMC12529835; doi:10.1186/s12904-025-01914-z)
Supplement: Supplementary file 1 — Supplementary Material 1. [file 12904_2025_1914_MOESM1_ESM.docx]

**COREQ (COnsolidated criteria for REporting Qualitative research) Checklist**

A checklist of items that should be included in reports of qualitative research. You must report the page number in your manuscript where you consider each of the items listed in this checklist. If you have not included this information, either revise your manuscript accordingly before submitting or note N/A.

| **Topic** | **Item No.** | **Guide Questions/Description** | **Reported on Page No.** |
| --- | --- | --- | --- |
| **Domain 1: Research team and reflexivity** | | | |
| *Personal characteristics* | | | |
| Interviewer/facilitator | 1 | Which author/s conducted the interview or focus group? | AV: primary moderator  NR: co-moderator |
| Credentials | 2 | What were the researcher’s credentials? E.g. PhD, MD | AV, NR and TS: Doctoral candidate, HJ: Dr., SSt and FH: Prof. Dr., ALB: PD Dr. |
| Occupation | 3 | What was their occupation at the time of the study? | AV: Academic researcher at the Carl von Ossietzky University in Oldenburg  NR: Academic researcher at the Carl von Ossietzky University in Oldenburg  TS: Academic researcher at the Medical School Hannover  HJ: Academic researcher at the Carl von Ossietzky University in Oldenburg  SSt: Professor for Health Services Research at the Medical School Hannover  FH: Professor for Health Services Research, Head of the Division of Outpatient Care and Pharmacoepidemiology at the Carl von Ossietzky University in Oldenburg  ALB: Head of the Junior Research Group on Rehabilitation Research at the Carl von Ossietzky University in Oldenburg |
| Gender | 4 | Was the researcher male or female? | AV: female, NR: female, TS: female, HJ: male, SSt: female, FH: male, ALB: female |
| Experience and training | 5 | What experience or training did the researcher have? | AV: qualitative research project, focus groups were used in the master thesis  NR: qualitative research project, focus groups were used in the master thesis  TS: experiences in the nursing home care and palliative care research  HJ: no experiences  SSt: much experiences through several research projects  FH: experiences in the nursing home care research  ALB: much experiences through several research projects |
| *Relationship with participants* | | | |
| Relationship established | 6 | Was a relationship established prior to study commencement? | p. 6  AV connected via the phone or via e-mail with most of the participants to ask for participation in the focus groups. NR had no contact to the participants before focus group interviews. TS, HJ, SSt, FH and ALB had no contact to the participants. |
| Participant knowledge of the interviewer | 7 | What did the participants know about the researcher? e.g. personal goals, reasons for doing the research | All participants got informed consent sheets before the focus groups started, where for example the reasons and aims of the focus groups were described. In the beginning of each focus group the moderators as well as the student assistant introduced themselves saying a few things about themselves (especially name and position in the project). |
| Interviewer characteristics | 8 | What characteristics were reported about the inter viewer/facilitator? e.g. Bias, assumptions, reasons and interests in the research topic | p. 21  No competing interests. |
| **Domain 2: Study design** | | | |
| Theoretical framework | | | |
| Methodological orientation and Theory | 9 | What methodological orientation was stated to underpin the study? e.g. grounded theory, discourse analysis, ethnography, phenomenology, content analysis | p. 7  The focus groups were analyzed, employing a deductive-inductive content analysis approach based on Kuckartz & Rädiker (2022). |
| *Participant selection* | | | |
| Sampling | 10 | How were participants selected? e.g. purposive, convenience, consecutive, snowball | p. 6  From May to September 2023, ACP facilitators were recruited for four focus groups. Recruitment occurred at an ACP networking meeting in Hannover (state capital of Lower Saxony) and through e-mail lists from hospice and palliative care associations, institutions offering further education and training in hospice and palliative care, organizations training ACP facilitators, all based in Lower Saxony, and the ACP Germany association. The practice advisory board of the Gut-Leben project also facilitated contact. |
| Method of approach | 11 | How were participants approached? e.g. face-to-face, telephone, mail, e-mail | p. 6  Interested participants were contacted by the research team directly by phone, e-mail, or in person, or could initiate contact with the study team by themselves. |
| Sample size | 12 | How many participants were in the study? | p. 8  24 ACP-facilitators |
| Non-participation | 13 | How many people refused to participate or dropped out? Reasons? | Not applicable. |
| *Setting* | | | |
| Setting of data collection | 14 | Where was the data collected? e.g. home, clinic, workplace | p. 6  The first three focus groups took place in person at the Carl von Ossietzky University of Oldenburg, while the fourth was held digitally using the university's web conferencing platform. |
| Presence of non-participants | 15 | Was anyone else present besides the participants and researchers? | One student assistant supported the conduction of the focus groups. |
| Description of sample | 16 | What are the important characteristics of the sample? e.g. demographic data, date | p. 8  The twenty-four participating ACP facilitators were on average 51.7 years old (range 30-70) and 75% were female (n=18). They completed their ACP training in the period from 2017 to 2022, so they have been working as ACP facilitators for different lengths of time. The nursing homes (NHs) in which the ACP facilitators work or have worked are 58.3% non-profit (n=14), 16.7% private (n=4), and public 4.2% (n=1), (not available n=5), and have a median of 188 beds, meaning the ACP facilitators work in NHs of varying sizes. |
| *Data collection* | | | |
| Interview guide | 17 | Were questions, prompts, guides provided by the authors? Was it pilot tested? | p. 6  The focus group guide was developed by the research team in collaboration with the Gut-Leben project's practice advisory board. First, the research team considered topics for the guide and prepared questions addressing the research question. In a second step, the draft of the focus group guide was discussed with the members of the practice advisory board. On the basis of this, the guide was adapted to the language used in the ACP practice, additional topics for the focus group guide were discussed, and the content sections were structured.  Focus group question guide, appendix. |
| Repeat interviews | 18 | Were repeat inter views carried out? If yes, how many? | No. |
| Audio/visual recording | 19 | Did the research use audio or visual recording to collect the data? | p. 7  With participant consent, the focus groups were audio-recorded and transcribed verbatim by an external professional transcription service following Fuß and Karbach (2019). All transcripts were pseudonymized. |
| Field notes | 20 | Were field notes made during and/or after the interview or focus group? | The moderating persons made some field notes while and after the focus groups. |
| Duration | 21 | What was the duration of the inter views or focus group? | p. 6  The four focus groups were conducted from July to September 2023, with an average duration of 115 minutes (range: 109 to 120 minutes). |
| Data saturation | 22 | Was data saturation discussed? | When planning the research project, the findings of the authors Hennink, Kaiser, and Weber (2019) were taken into account, who describe in their article that code saturation can be achieved after four focus group discussions. This was considered when conducting this study with N=4 focus groups.  Hennink MM, Kaiser BN, Weber MB. What Influences Saturation? Estimating Sample Sizes in Focus Group Research. Qualitative Health Research. 2019;29(10):1483-1496. doi:[10.1177/1049732318821692](https://doi.org/10.1177/1049732318821692) |
| Transcript returned | 23 | Were transcripts returned to participants for comment and/or correction? | No. |
| **Domain 3: analysis and findings** | | | |
| Data analysis | | | |
| Number of data coders | 24 | How many data coders coded the data? | p. 7  Two persons coded the data. |
| Description of the coding tree | 25 | Did authors provide a description of the coding tree? | p. 8  appendix |
| Derivation of themes | 26 | Were themes identified in advance or derived from the data? | p. 7  deductive (main categories) – inductive (fine coding subcategories) |
| Software | 27 | What software, if applicable, was used to manage the data? | p. 7  MAXQDA |
| Participant checking | 28 | Did participants provide feedback on the findings? | No. |
| *Reporting* | | | |
| Quotations presented | 29 | Were participant quotations presented to illustrate the themes/findings? Was each quotation identified? e.g. participant number | p. 8 – p. 14 |
| Data and findings consistent | 30 | Was there consistency between the data presented and the findings? | p. 8 – p. 14 |
| Clarity of major themes | 31 | Were major themes clearly presented in the findings? | p. 8 – p. 14 |
| Clarity of minor themes | 32 | Is there a description of diverse cases or discussion of minor themes? | p. 8 – p. 14 |

Developed from: Tong A, Sainsbury P, Craig J. Consolidated criteria for reporting qualitative research (COREQ): a 32-item checklist

for interviews and focus groups. International Journal for Quality in Health Care. 2007. Volume 19, Number 6: pp. 349 – 357

Once you have completed this checklist, please save a copy and upload it as part of your submission. DO NOT include this

checklist as part of the main manuscript document. It must be uploaded as a separate file.
