# Supplementary material for: Advance care planning in German nursing homes from the perspective of the facilitators: A focus group study
Source: BMC Palliat Care. 2025 Oct 15;24:258. doi: 10.1186/s12904-025-01914-z (PMC12529835; doi:10.1186/s12904-025-01914-z)
Supplement: Supplementary file 2 — Supplementary Material 2. [file 12904_2025_1914_MOESM2_ESM.docx]

| Main categories | Subcategories | Definition of the categories | Anchor example |
| --- | --- | --- | --- |
| Consultation Process | **Getting in touch** | Text passages describing the point of (initial) contact were marked, and their barriers and supporting factors. | “Everyone who moves in gets a flyer handed to them.” [FG3]  “The residents themselves actually don’t really approach me that often.” [FG2]  “I noticed something else too — I’m not the one who gets the initial trust in the facility, that trust goes to the care staff. Or the people who actually work there. So that really showed me how important it is to involve the nurses and the team, because they’re the ones who really connect with the residents.” [FG4] |
|  | **Conversation procedure** | Text passages were marked that describe the conversation procedure, and their barriers and supporting factors. | “So, I mainly have the conversations directly with the resident. Sometimes they say, like, 'my son' or someone should be there. That’s fine with me. Otherwise, I often finish the conversations alone with the resident.” [FG2]  “Sometimes there are things/residents where you have to go five or six times. With some, after just two times, it’s clear and solid on paper, you know?” [FG2]  “In a way, yes. But also, not really. Like [B1] says, every conversation has its basic flow. But at the same time, each one is totally unique.” [FG3] |
|  | **Documentation** | Text passages containing the documentation of care preferences or related to the provision of documents were marked, and their barriers and supporting factors. | “And then the document goes to the general practitioner. Then I have a brief chat with her, so to speak, and then she stamps it and signs it.” [FG1]  “And then I upload it into the system. Of course, the original goes back to the person. A copy goes to the care department, and a scanned file is sent to the administration, who then upload it into this SIS [Note: abbreviation for Structured Information Collection, care documentation system]." [FG1]  “Nineteen pages, my colleague complains every time she has to print a transfer form for the hospital. (speaks with a changed voice) 'I don't have that much paper in there, and it takes so long.' They don't want to wait that long. We need to find more practical solutions." [FG1] |
|  | **Billing** | Text passages dealing with billing were coded, and their barriers and supporting factors. | “For example, that was a problem when you don't have a contact person. Then I once tried to call an insurance company, you can take any as an example, it doesn't matter: “No, I don't know it. Never heard of it”.” [FG1]  “I also had the experience, so we had agreed on the lump sum remuneration for 2020. During the first conversations, I filled out the service records. Sent it off. Got no feedback. I contacted them via email, the person had no idea, because it's actually under §132g, and we don't need it. You get a lump sum remuneration." [FG1] |
| ACP facilitators | **Career history** | Text passages describing the career history of the facilitators were marked. | “In 2019, I did the BVP training as a conversation facilitator in [place].” [FG1]  “I’ve been working in home management in elderly care since 1990, and since 2020, I’ve been responsible for introducing the GVP project in our facilities.” [FG2] |
|  | **Job description** | Text passages have been marked that refer to the job description. | “Yeah, I actually work full-time as a nurse in elderly care, in a nursing home, during the day shift. And then I have a certain hourly rate per hour [proportion of working hours] for ACP.” [FG2]  “So, at the beginning, it was honestly a lot, and I worked behind the scenes at first. I trained the whole team, even up to the palliative care sector. They also have specialists in palliative care because it’s all connected, as we all know. So, basically, I had to train everyone on-site first, including all the processes and structure—training, training, training, and more training. Then there were the conversations with the residents, the families, and so on. It was quite a package because, in the beginning, I was the solitary fighter." [FG2] |
