# Supplementary material for: Advance care planning in German nursing homes from the perspective of the facilitators: A focus group study
Source: BMC Palliat Care. 2025 Oct 15;24:258. doi: 10.1186/s12904-025-01914-z (PMC12529835; doi:10.1186/s12904-025-01914-z)
Supplement: Supplementary file 3 — Supplementary Material 3. [file 12904_2025_1914_MOESM3_ESM.docx]

**Guiding questions for the focus groups**

___________________________________________________________________________

**Introduction:**

A brief introduction of yourself and description for us which facilities you work in and how long you have been offering ACP.

___________________________________________________________________________

**Topic block 1:** *Description of the day-to-day of ACP*

How would you describe your work in the context of ACP?

__________________________________________________________________________

**Topic block 2:** *ACP process/procedure*

What is the overall course of an ACP counseling process (from establishing contact, the first conversation, and documenting the content of the conversation following further counseling sessions)?

__________________________________________________________________________

**Topic block 3:** *Supporting factors and barriers, as well as suggestions for improvement*

Can you tell us from your experience what an ACP process looks like when it runs smoothly?

__________________________________________________________________________
