# Supplementary material for: Advance care planning in German nursing homes from the perspective of the facilitators: A focus group study
Source: BMC Palliat Care. 2025 Oct 15;24:258. doi: 10.1186/s12904-025-01914-z (PMC12529835; doi:10.1186/s12904-025-01914-z)
Supplement: Supplementary file 4 — Supplementary Material 4. [file 12904_2025_1914_MOESM4_ESM.docx]

# German References – Further Information

| **Reference** | **Comment** |
| --- | --- |
| Schwinger A, Jürchott K, Behrendt S, Argüello Guerra F, Stegbauer C, Willms G, Klauber, J. Krankenhausaufenthalte von Pflegeheimbewohnenden am Lebensende: Eine empirische Bestandsaufnahme. In: Jacobs K, Kuhlmey A, Greß S, Klauber J, Schwinger A (eds). Pflege-Report 2022: Spezielle Versorgungslagen in der Langzeitpflege. Berlin, Heidelberg: Springer; 2022. S. 53–74. doi: 10.1007/978-3-662-65204-6_4. | An English summary of this German source can be found via the link <https://link.springer.com/chapter/10.1007/978-3-662-65204-6_4> (book PDF, p. 54).  *“Nursing homes are important providers of end-of-life care: one out of three insurees of a German regional statutory health insurance fund (AOK) who died within one year lived in a nursing home. Although the legal framework for palliative care has changed considerably in the last 15 years, the challenges for nursing homes caring for the dying are immense. This paper presents the key findings of a claims data analysis of hospital transfers of nursing home residents at the end of their lives. It can be shown that hospitalisation increases near the end of life and is associated with ambulatory care sensitive conditions. The paper aims to highlight this issue of potentially unmet needs and to initiate a discussion of what changes are needed to ensure timely and consistent recording of the wishes of nursing home residents with regard to their end-of-life care.”* |
| Dasch B, Lenz P. [Place of death of older people with dementia Epidemiological data from an observational study of places of death in Germany (2001, 2011, 2017)]. Z Gerontol Geriatr. 2022; 55 (8): 673–679. doi: 10.1007/s00391-021-01976-7. | An English title and an English abstract are available.  <https://link.springer.com/article/10.1007/s00391-021-01976-7> |
| Derler F. [Human dignity and autonomy in medicoethical decisions at the end of life]. Z Gerontol Geriatr. 2024; 57: 550–555. doi: 10.1007/s00391-024-02308-1. | An English title and an English abstract are available.  <https://link.springer.com/article/10.1007/s00391-024-02308-1> |
| GKV-Spitzenverband. Vereinbarung nach § 132g Abs. 3 SGB V über Inhalte und Anforderungen der gesundheitlichen Versorgungsplanung für die letzte Lebensphase vom 13.12.2017; 2017. https://www.dhpv.de/files/public/themen/2018_Vereinbarung_nach_132g_Abs_3_SGBV_GVP.pdf. Accessed 13 Feb 2025. | The agreement contains more detailed information on the content and requirements of health care  planning for the last phase of life in accordance with §132g SGB V. The agreement regulates the objectives of the ACP service, the group of people entitled to it, the qualifications of the ACP facilitators as well as the requirements, organization, documentation and financing of ACP for those insured by the statutory health insurances. |
| GKV-Spitzenverband. Bericht des GKV-Spitzenverbandes zur Palliativversorgung: Bericht des GKV-Spitzenverbandes zum Stand der Entwicklung sowie der vertraglichen Umsetzung der Spezialisierten ambulanten Palliativversorgung (SAPV), der allgemeinen ambulanten Palliativversorgung im Rahmen der häuslichen Krankenpflege sowie der gesundheitlichen Versorgungsplanung für die letzte Lebensphase. Berlin: GKV-Spitzenverband; 2023. https://www.gkv-spitzenverband.de/media/dokumente/krankenversicherung_1/hospiz_palliativversorgung/20231211_Bericht_SAPV_HKP_und_132g.pdf. Accessed 27 May 2025. | The report by the National Association of Statutory Health Insurance Funds summarizes the legally prescribed development and implementation of palliative care in Germany. It is based on various German laws on hospice and palliative care, e.g. ACP. The report documents the status of contractual implementation of these services as at the reporting date of 31.12.2022, broken down into SAPV, home nursing care and care planning. The aim is to provide an overview of the care situation and its further development. |
| Fuß S, Karbach U. Grundlagen der Transkription: Eine praktische Einführung. 2. Auflage. Stuttgart, Opladen, Toronto: utb; 2019. ISBN 978-3-8252-5074-4. | This German book by Susanne Fuß and Ute Karbach is a practical guide to transcribing scientific interviews for qualitative social research. The volume presents the basics of transcription and its application as well as the rules of transcription. Transcription means that collected data (e.g. audio data from interviews or focus groups) is written down. By transcribing, the spoken word and, if applicable, the phonetic expression are literally transferred. This transcription of the spoken word into written language makes the interview and group discussion available for evaluation in the form of written data. |
| Kuckartz U, Rädiker S. Qualitative Content Analysis: Methods, Practice and Software. 2nd Edition. Los Angeles; London; New Delhi; Singapore; Washington DC; Melbourne: SAGE; 2023. ISBN 9781529609134. | Replaced by an English-language source from the same authors as before. (ISBN 9781529609134) |
| Stadelmann KA. Das Lebensende in der (heutigen) Gesellschaft. In: Stadelmann KA (eds). Die Sorge um Andere am Lebensende als Beruf. Sozialwissenschaftliche Gesundheitsforschung. Wiesbaden: Springer FVS Wiesbaden; 2024. S. 19–28 (Sozialwissenschaftliche Gesundheitsforschung). doi: 10.1007/978-3-658-45031-1_2. | This source is a chapter in an edited book. This chapter reviews the current state of research on social work in the field of dying and death and in palliative care - based on the literature research conducted specifically for this purpose, which was carried out between 2018 and 2021 in particular and supplemented with more recent publications from 2021 to March 2023. The presentation of the state of research therefore reflects the current state of the scientific debate in these years and addresses German-language and some international publications, but does not claim to be exhaustive.  <https://link.springer.com/book/10.1007/978-3-658-45031-1> |
| Hartog CS, Spies CD, Michl S, Janssens U. [Advance care planning during the coronavirus pandemic-A chance for patient autonomy in acute situations]. Med Klin Intensivmed Notfmed. 2020; 115 (7): 571–572. doi: 10.1007/s00063-020-00717-9. | An English title and an English abstract are available.  <https://link.springer.com/article/10.1007/s00063-020-00717-9> |
| Jacobs H, Brütt AL, Völkel A, Stiel S, Schleef T, Schütte S et al. [Feasibility study and analyses of the service records for advance care planning (ACP according to sect. 132g SGB V) in nursing homes - Results of the 'Gut-Leben' project]. Z Evid Fortbild Qual Gesundheitswes. 2024; 190-191: 84–91. doi: 10.1016/j.zefq.2024.10.006. | An English title and an English abstract are available.  <https://www.sciencedirect.com/science/article/pii/S186592172400223X?via%3Dihub> |
| Schnakenberg R, Fischer R, Kreutzberg U, Gerdes V, Wiemann M, Schmidtkunz J et al. [Inventory and Needs Assessment for the Implementation and Networking of Advance Care Planning According to §132 g SGB V in Lower Saxony – Results of an Online Survey of Facilitators]. Z Palliativmed. 2023; 24 (03): 129–137. doi: 10.1055/a-2036-3066. | An English title and an English abstract are available.  <https://www.thieme-connect.de/products/ejournals/abstract/10.1055/a-2036-3066> |
| Voß H, Kruse A. [Advance care planning (ACP) in the context of dementia: Development of an instrument for exploration of perspectives of affected persons]. Z Gerontol Geriatr. 2019; 52 (Suppl 4): 282–290. doi: 10.1007/s00391-019-01624-1. | An English title and an English abstract are available.  <https://link.springer.com/article/10.1007/s00391-019-01624-1> |
| Statistisches Bundesamt (Destatis). Pflegestatistik 2019. Pflege im Rahmen der Pflegeversicherung: Deutschlandergebnisse – 2019; 2020. https://www.statistischebibliothek.de/mir/servlets/MCRFileNodeServlet/DEHeft_derivate_00074028/5224001199004.pdf. Accessed 20 Feb 2024. | The care statistics have been compiled by the Federal and State Statistical Offices every two years since December 1999. The aim of the statistics is to obtain data on the supply of and demand for nursing care. It therefore collects data on those in need of care and on nursing homes and outpatient services, including staff. <https://www.statistischebibliothek.de/mir/servlets/MCRFileNodeServlet/DEHeft_derivate_00074028/5224001199004.pdf> |
